# Sustained mental health outcomes from the youth readiness intervention: A four‐year effectiveness follow‐up of a hybrid type II trial in Sierra Leone

**DOI:** 10.1111/jcpp.70158

**Published:** 2026-04-27

**Authors:** Wijnand Van Den Boom, Kathryn Noon, Shuangshuang Yang, Renee Menart, Fatoma Momoh, Abdulai Jawo Bah, Matias Placencio‐Castro, Theresa S. Betancourt

**Affiliations:** ^1^ Research Program on Children and Adversity, School of Social Work Boston College Chestnut Hill MA USA; ^2^ Innovation for Poverty in Action New Haven CT USA

**Keywords:** Youth Readiness Intervention, cognitive‐behavioral therapy, entrepreneurship training, implementation science, hybrid type II cluster‐randomized trial, mental health, emotion regulation, daily functioning, Sierra Leone

## Abstract

**Background:**

Strategies to expand access to care and sustain evidence‐based mental health interventions (EBIs) must be tested within novel delivery platforms to extend the reach of services in fragile and conflict‐affected settings. Integration into broader development programs may help maintain long‐term effects. This study presents a four‐year follow‐up of a previously conducted Hybrid Type II implementation‐effectiveness cluster‐randomized trial (CRT) of the Youth Readiness Intervention (YRI)—an EBI drawing on cognitive‐behavioral, interpersonal, and mindfulness‐based approaches—delivered within a youth entrepreneurship program in Sierra Leone (2018–2019).

**Methods:**

Long‐term mental health outcomes (emotion regulation, psychological distress, and interpersonal functioning) were examined among a randomly selected subgroup of 584 participants across three study arms: a control group, a group that only received entrepreneurship training (‘ENTR’), and a group that received both YRI and ENTR (‘YRI + ENTR’). Linear mixed‐effect models accounted for the nested structure of the data. Impact of the COVID‐19 pandemic was also assessed through economic and relationship stressors using structural equation modeling.

**Results:**

Four years postintervention, YRI + ENTR‐youth maintained improvements in depression (*β* = −.057; 95% CI −0.09 to −0.02; [effect size] *d* = −.111) and combined depression/anxiety symptoms (*β* = −.047; 95% CI −0.08 to −0.01; [effect size] *d* = −.096) though no sustained effects were observed for daily functioning or emotion regulation. No differences were found for ENTR‐only participants versus controls and YRI + ENTR. COVID‐19‐related economic stressors mediated the relationship between study arm and mental health, revealing small but significant effects.

**Conclusions:**

In low‐income settings like Sierra Leone, where formal mental health services are scarce, sustainable community‐based interventions such as the combined YRI + ENTR intervention offer a critical approach to reducing psychological distress. Sustained long‐term benefits suggest that participants developed coping strategies that supported resilience during challenges like the COVID‐19 pandemic. These gains may also foster broader community resilience, enhancing both individual well‐being and collective capacity to withstand future adversity.

## Introduction

The consequences of violence and compounded adversity among youth in conflict‐affected countries in sub‐Saharan Africa contribute to high rates of depression, anxiety, and posttraumatic stress reactions (Betancourt, Thomson, & VanderWeele, 2018), alongside reduced labor participation and quality of life (Patel et al., 2018). However, access to mental health services remains limited, with most low‐ and middle‐income countries allocating <2% of their health budgets to mental health (Moitra et al., 2022). In fragile, conflict‐affected settings, the treatment gap often exceeds 75% (Vos et al., 2020). In Sierra Leone, multiple crises—including an 11‐year civil war, the Ebola outbreak, climate‐related disasters, and the COVID‐19 pandemic—have strained the country's health, social, and mental health systems, severely limiting service capacity (Amnesty International, 2021; Richards, 2020).

A shortage of trained mental health professionals (Betancourt, Meyers‐Ohki, Charrow, & Tol, 2013) exacerbates the treatment gap in Sierra Leone (Bond et al., 2022). To address this, as a part of National Institutes of Mental Health (NIMH) global implementation science research hubs (Collins, Price, & Pringle, 2014), the Youth Functioning and Organizational Success for West African Regional Development (Youth FORWARD) initiative was launched in 2017 to integrate the Youth Readiness Intervention (YRI) into entrepreneurship programs in Sierra Leone (Betancourt et al., 2021). The YRI is a transdiagnostic, evidence‐based group mental health intervention consisting of 12 modules. It uses common practice therapeutic components drawn from cognitive‐behavioral, interpersonal therapy, and mindfulness techniques delivered by nonspecialists and integrated into alternative delivery platforms. The Youth FORWARD initiative in partnership with the Sierra Leone Ministry of Youth Affairs and the German development agency *Deutsche Gesellschaft für Internationale Zusammenarbeit* (GIZ) tested a Collaborative Team Approach (CTA) as a strategy to enhance quality of delivery of the YRI by nonspecialists who also led entrepreneurship training to youth ages 18–30 across three rural districts.

.Guided by the EPIS (Exploration, Preparation, Implementation, Sustainment) framework (Aarons, Hurlburt, & Horwitz, 2011), phased integration under the CTA strategy adapted the YRI to rural contexts, while also setting up Plan–Do–Study–Act (PDSA) problem‐solving cycles (Taylor et al., 2014). PDSA is an iterative, data‐driven approach that involves planning changes, implementing them on a small scale, studying the outcomes, and acting on the results to refine and improve an intervention. This ensures that any implementation barriers (such as poor attendance) are overcome promptly and that feedback loops of testing strategies to overcome implementation barriers are initiated. Youth FORWARD effectiveness data under this delivery strategy was conceptualized as a Hybrid Type II implementation‐effectiveness study, which examined the impact of the CTA on fidelity and competence while an embedded cluster‐randomized trial examined mental health and functioning outcomes to assess the intervention's effectiveness.

The pre‐to‐post Youth FORWARD trial found improvements in depression and anxiety symptoms among YRI participants who also participated in the entrepreneurship program, along with high feasibility and acceptability when the intervention was delivered by nonspecialists also working within the entrepreneurship program (Freeman et al., 2024). Compared to those in the control group, community leaders observed improvements in youth behaviors (e.g., cooperation in group efforts) and overall performance (e.g., ability to complete tasks on time) among YRI + entrepreneurship program participants, and youth themselves reported moderate to high acceptability of the YRI.

The present paper reports the four‐year follow‐up of the Youth FORWARD Hybrid Type II trial, focusing specifically on the potentially sustained long‐term effectiveness of the YRI on mental health, while implementation outcomes based on baseline data are presented elsewhere (Freeman et al., 2024). Analyses draw on a randomly selected subsample of youth from the original trial, including participants from both intervention and control groups, and examine sustained mental health effects observed in this cohort. Longer‐term follow‐up is crucial to provide valuable insights into the durability of interventions, helping to refine and improve future programs, and adding to our understanding of potential return on investment (Tol et al., 2011). While youth mental health interventions in sub‐Saharan Africa have shown significant potential, most only achieve short‐term success due to factors such as socioeconomic challenges, ongoing trauma, or lack of continued support to beneficiaries (Mabrouk et al., 2022).

Adding significant challenges to an already difficult environment, the global COVID‐19 pandemic hit Sierra Leone between the last follow‐up in 2019 and the current follow‐up in 2023 leading to stay‐at‐home orders and economic shutdowns. As a part of understanding the ecosystem for implementation informed by the EPIS model, the present analysis also investigates the potential effects of the pandemic on both youth mental health and economic functioning across the study arms. Specifically, we will examine whether changes in family relationships and economic conditions during the pandemic mediate mental health outcomes, as these disruptions may have influenced the long‐term effectiveness of the YRI. Understanding contextual factors occurring within the ecosystem is key to evaluating the viability of the EBI for sustainment within alternative delivery platforms and arguments that can be made about return on investment.

## Methods

### Original CRT study design

The original study design entailed recruiting youth based on prespecified inclusion criteria (i.e., 18–30 years of age, disengaged from school, not pregnant nor working full time, and having elevated scores on measures of emotion regulation and functioning). According to the Government of Sierra Leone, the official definition of youth includes individuals aged 15–35 years (Alemu, 2016). This age range for youth in Sierra Leone reflects the socioeconomic realities and cultural understanding of the transition from adolescence to full adulthood in the country. Geographic clusters of eligible youth were created (stratified by age and sex) and matched into triads based on youth and geographic characteristics to minimize bias. Once matched, the clusters were randomly assigned to one of three groups: ‘YRI + ENTR’ (receiving both mental health and entrepreneurship interventions), ‘ENTR’ (receiving only the entrepreneurship intervention), or ‘Control’ (receiving neither intervention). Group allocations of the participants and the data collectors were both masked. Clusters that could not be matched were treated as standalone localities. Details on the matching procedure and the design of the cluster‐randomized trial, including the randomization process, can be found elsewhere (Betancourt et al., 2021; Freeman et al., 2024).

### Sample selection

A two‐step stratified random sampling approach was used. Clusters were randomly identified and rank‐ordered, with stratification by district and study arm to ensure balanced representation. Individuals were further stratified by gender and cluster, rank‐ordered using a random number generator, and assigned to main or replacement groups for recontact. The research agency (IPA Sierra Leone) received a dataset with participant IDs, geographic data, sample status, and rank order. Selection began with the first main cluster per district; within each, the first male and female main subjects were contacted. Replacements were contacted only after main participants were exhausted. When all main clusters were used, replacement clusters were activated. Over 78% of participants were main subjects.

### Youth Readiness Intervention

The YRI is a 12‐module, transdiagnostic mental health intervention drawing on cognitive‐behavioral, interpersonal, and mindfulness‐based therapies (Newnham et al., 2015). It was delivered by nonspecialists who also facilitated the entrepreneurship program, as part of the Youth FORWARD initiative implemented in 2019. Implementation followed the Collaborative Team Approach (CTA), using Plan–Do–Study–Act cycles to address delivery challenges and adapt the intervention to local contexts, for example, accommodating agricultural schedules in Koinadugu (Betancourt et al., 2014). Facilitator competence and fidelity were monitored weekly, using standardized YRI observation checklists and competence rating scales, completed by YRI Seed Team supervisors through in‐person and remote observation, to ensure quality (see Freeman et al., 2024 for full implementation results). YRI Seed Team members trained and supervised facilitators through in‐person observation and remote support. The same facilitators also delivered a 15‐session entrepreneurship training tailored to rural youth with low literacy. Attendance data were available for approximately half of the Youth FORWARD cohort, among whom participants completed an average of 11.6 YRI sessions, with 96% completing all core modules—indicating strong adherence and fidelity to intervention delivery. In Youth FORWARD, the YRI was delivered prior to entrepreneurship training, with assessments at baseline and postentrepreneurship to assess short‐term effects (Betancourt et al., 2021). The current study represents the four‐year follow‐up of a subsample of these Youth FORWARD participants to examine the longer‐term effects of the combined YRI + entrepreneurship program.

### Mental health outcomes

Mental health outcomes reported here repeat those examined in a previous measurement of the Youth FORWARD cohort (Freeman et al., 2024). Emotion regulation was assessed using an adapted version of the 36‐item Difficulties in Emotion Regulation Scale (DERS), with response options ranging from [1] ‘almost never’ to [5] ‘almost always’ (Roszkowski & Soven, 2010). To make the scale suitable for the Sierra Leonean context and improve its psychometric properties, negatively worded items were reworded. A summative score was calculated for each participant, with higher overall scores reflecting poorer emotional regulation. Interpersonal and daily functioning impairment was measured with the World Health Organization Disability Assessment Schedule (WHODAS) 12‐item short form, with response options ranging from [0] ‘none’ to [5] ‘extreme or can't do’ (Üstün, 2010). Functional impairment was measured across five different domains: understanding and communicating, mobility, self‐care, life activities, and participation in society. A summative score was calculated with higher scores reflecting higher functional impairment. Anxiety and depression symptoms were assessed using the 25‐item Hopkins Symptom Checklist (HSCL‐25), with response options ranging from [1] ‘not at all’ to [4] ‘extremely’ (Hesbacher, Rickels, Morris, Newman, & Rosenfeld, 1980). The HSCL‐25 has shown good internal reliability in Sierra Leone (Betancourt, Borisova, De La Soudière, & Williamson, 2011). Total symptom score was calculated by averaging all 25 items. Additionally, separate mean scores for the anxiety and depression subscales were calculated to examine domain‐specific symptom severity. Higher scores on each subscale reflect more severe symptoms.

### COVID‐19 related variables

Participants were asked about the perceived changes in family relationship dynamics since the COVID‐19 pandemic. We computed a composite measure that included four items that assess different aspects of these relationships: the amount of time spent together, the frequency and quality of conversations, the ability to face challenges together, and the level of emotional support or concern shown between individuals. Each item was measured on a 5‐point response scale ranging from [1] ‘decreased a lot’ to [5] ‘increased a lot’, with higher scores indicating better relationship quality. A mean score was computed across these four items, yielding a Cronbach's alpha of .87. In addition, we assessed the economic challenges faced by households during the COVID‐19 pandemic, comprising four key aspects: losing the main source of income (yes, completely/yes, partially/no), changes in income (no/yes), reduced working hours (no/yes), and job opportunities within the household (no/yes). Each item was recoded so that higher scores indicated better economic outcomes. A composite mean score was calculated across these four variables, with higher scores representing better economic conditions, yielding a Cronbach's alpha of .81.

### Statistical analyses

Descriptive data were summarized by means and standard deviations for continuous variables, frequencies, and proportions for categorical variables. To test for mental health effectiveness and to account for the nested structure of our data—specifically, timepoints nested within participants (level 1), and participants nested within clusters (level 2)—linear mixed‐effects models were used. The modeling strategy involved incorporating fixed effects for time (baseline vs 4‐year follow‐up), study arm (control vs ENTR vs YRI + ENTR), and their two‐way interaction. In addition, participant characteristics, including age at baseline, sex, reading skills, writing skills, and educational level, as well as district location and a unique identifier for ‘standalone’ clusters, were included in the model as covariates to control for potential confounding effects.

District was included as a fixed effect to account for differences derived from geographical accessibility and access to services at a level that was not necessarily captured by the cluster‐level matching variables. This was particularly important because districts vary in factors such as accessibility to healthcare, infrastructure, or other services that could impact participants' mental health outcomes or intervention effects. Furthermore, the standalone marker variable was included to account for potential confounding variance coming from clusters that did not have a strict match across the intervention groups. Due to remaining geographical dissimilarities across intervention districts after randomization at baseline, and to address any potential imbalances following the matching of clusters during randomization to treatment and control groups, five components extracted from cluster‐level variables were included as fixed effects in the models, along with their interactions with the district, as was previously done (Freeman et al., 2024).

For each outcome, null models without covariates and with random intercepts for cluster and participant were used to obtain the standard deviations for the random effects. From the final models, which included all potential confounders, we extracted the unstandardized regression coefficient (*b*) and the standard error for the interaction between study arm and time point (continuous). To obtain standardized effect sizes, these parameters, along with sample sizes for treatment and control, and intra‐class correlation (ICC, which measures the proportion of total variance attributable to between‐group differences), were then entered into a web‐based effect size calculator (Wilson, 2023).

In our models, the levels of nesting included time at level 1 (within‐subject repeated measures) and gender‐specific design groups at level 2 (clusters). The within‐subject ICCs (level 1) ranged from .13 to .18, which falls within the commonly referenced range of .1 to .25 in multilevel modeling literature. This indicated a moderate proportion of variability in outcomes within individuals across timepoints. This is consistent with expectations for repeated‐measures designs, where individuals are subject to developmental changes, environmental influences, and intervention effects over time. The ICCs for the design group level ranged from .05 to .08 and reflected a small proportion of variability attributable to clustering, likely due to shared contextual factors such as social norms, environment, or access to services.

Our modeling approach helps prevent the underestimation of standard errors and overstatement of statistical significance. By including ‘design group’ (cluster) as a random effect, we take into account the similarities among participants within the same group, which is especially important because our study used a cluster‐randomized design, where groups rather than individuals were assigned to conditions (e.g., arms were stratified by gender). Importantly, this approach aligns with the longitudinal cluster‐randomized design of the study, where such multilevel models are necessary to accurately capture within‐ and between‐group variation across timepoints (Betancourt et al., 2021). It allows us to capture important patterns of change within individuals and account for the small, but present, similarities among participants within the same group.

Analyses were conducted using data from all participants who completed both baseline and four‐year follow‐up assessments (*n* = 1,151), as well as those who were randomly selected to participate at the four‐year follow‐up (*n* = 584). Missing data points were addressed using a chained equations approach that allowed for imputation at the item level (Plumpton, Morris, Hughes, & White, 2016). Ten complete datasets were generated and analyzed. Imputation models accounted for the longitudinal nature of the data and included participants' outcome scores as well as demographics (age, sex, and educational level), study arm, and cluster. Overall levels of missingness at the four‐year follow‐up were low, ranging from 0% to .86% across outcome measures.

To investigate the mediation effect of COVID‐19 burden (i.e., perceived changes in family relationships and economic challenges due to the COVID‐19 pandemic) in the relationship between study arm and mental health outcomes over time, we employed a structural equation modeling (SEM) approach. SEM was chosen for its ability to model complex relationships and test both direct and indirect effects simultaneously, making it ideal for examining mediation processes. The model examined both the direct and indirect effects of the interaction between the study arm and time point on the outcome variables. The COVID‐19 variables were hypothesized to partially mediate the effect of this interaction on mental health outcomes by capturing the burden of COVID‐19. By including the interaction between study arm and time points, the model allowed for capturing differential effects of the intervention over time. We conducted the analysis using maximum likelihood estimation and tested the significance of both direct and indirect effects. Missingness was addressed using full information maximum likelihood (FIML), a method that provides more efficient and unbiased parameter estimates by utilizing all available data points, assuming the data are missing at random. FIML was selected to maximize the robustness of our analysis despite the presence of missing data. To ensure the robustness of our findings, we calculated standard errors using the observed information matrix, and further validated the results through bootstrapping, assessing significance via z‐tests. These analyses were performed on the subsample of participants at the four‐year follow‐up data point.

Any *p*‐value lower than .05 was considered statistically significant (two‐tailed). All analyses were conducted in Stata version 18 (StataCorp LLC, College Station, TX).

## Results

Participants were 584 Sierra Leonean youth from three rural districts in Sierra Leone (Kailahun, Koinadugu, and Kono) who were randomly selected from the original Youth FORWARD sample of 1,151 youth that participated in the baseline assessment in 2019. Interviews were done between May 2023 and October 2023. The number of clusters within each study arm ranged between 19 and 21, with an average cluster size of 9–11 participants. Participants had a mean age of 24.8 (range 18–30 years), 47.4% were female, 74.4% had ever attended school, and 84.0% were married or had an intimate partner. Baseline characteristics were similar across the three study arms, except for district (Table 1). The four‐year follow‐up sample (*n* = 584) showed sociodemographic characteristics similar to those of the original sample (*n* = 1,151).

**Table 1:** Baseline characteristics of the study sample (*N* = 584)

	Study arm			*p* *
	Control	ENTR only	YRI + ENTR	
	*N* = 189 (32.4%)	*N* = 201 (34.4%)	*N* = 194 (33.2%)	
Age, mean (*SD*)	24.8 (3.5)	24.8 (3.4)	24.9 (3.5)	.799
Sex				
Male	105 (55.6%)	99 (49.3%)	98 (50.5%)	.424
Female	84 (44.4%)	102 (50.7%)	96 (49.5%)	
Educational level				
None/Some primary	84 (44.4%)	85 (42.3%)	85 (43.8%)	.906
Primary or beyond	105 (55.6%)	116 (57.7%)	109 (56.2%)	
Reading skills				
Cannot read	85 (45.0%)	85 (42.3%)	93 (47.9%)	.529
Can read	104 (55.0%)	116 (57.7%)	101 (52.1%)	
Writing skills				
Cannot write	69 (36.5%)	72 (35.8%)	76 (39.2%)	.769
Can write	120 (63.5%)	129 (64.2%)	118 (60.8%)	
District				
Kailahun	69 (36.5%)	60 (29.9%)	78 (40.2%)	.031
Koinadugu	46 (24.3%)	72 (35.8%)	61 (31.4%)	
Kono	74 (39.2%)	69 (34.3%)	55 (28.4%)	

* Chi‐square test.

Descriptive statistics for all primary outcome variables (depression, anxiety, daily functioning, and emotion regulation) are presented in Table S1. These values provide context for the overall levels of distress and functioning among youth at baseline and follow‐up.

**Figure 1:**
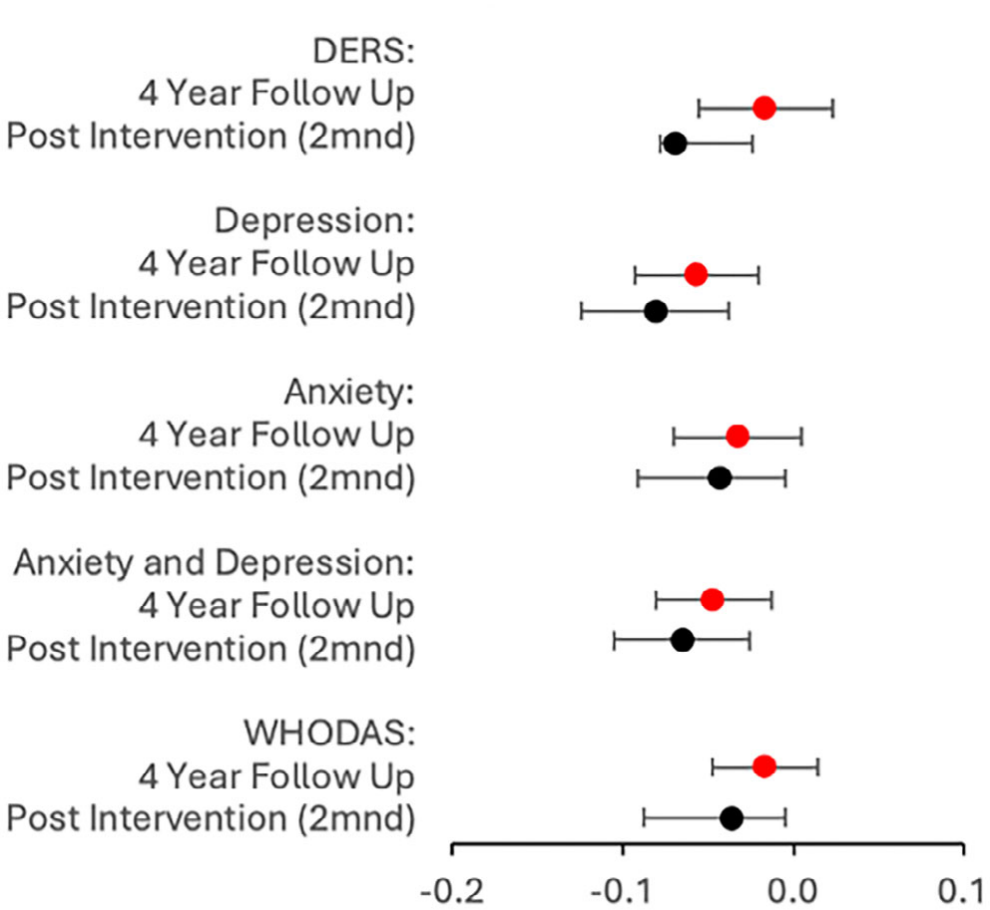
Youth mental health intervention effectiveness estimates from mixed‐effects models, *n* = 584 youth, YRI follow‐up (2023) versus postintervention (2018/2019). Red nodes represent current results from linear mixed models comparing four‐year follow‐up versus baseline (*n* = 584). Black nodes represent the results from linear mixed models comparing 2 months postintervention versus baseline (*n* = 1,151) (Freeman et al., 2024). Each node represents the effect of YRI + ENTR versus control in influencing mental health, with the beta coefficients (*ß*) indicating the direction and strength of these associations. A positive beta (*ß* > 0) suggests a decline in mental health; a negative beta (*ß* < 0) indicates an improvement in mental health. DERS, Difficulties in Emotion Regulation Scale; WHODAS, World Health Organization Disability Adjustment Scale. Depression and Anxiety were measured with the Hopkins Symptom Checklist

Results from the linear growth models are presented in Figure 1. Improvements in combined total anxiety/depression symptoms (*ß* = −.047; 95% CI = −0.08 to −0.01, *d* = −.096), and depression symptoms (*ß* = −.057; 95% CI = −0.09 to −0.02, *d* = −.111) were observed for the YRI + ENTR group compared to the control group. For anxiety symptoms, the YRI + ENTR group had a sustained reduction in anxiety (*ß* = −.033; 95% CI = −0.07 to 0.00, *d* = −.062), though the confidence interval suggests this effect is close to the threshold of statistical significance. There was no evidence of improvements in daily functioning or emotion regulation when comparing the YRI + ENTR group to the control group. Importantly, ENTR‐only participants did not differ from controls on any mental health outcomes. Furthermore, there were no differences in mental health outcomes when comparing YRI + ENTR directly to ENTR (Table S2).

Other main fixed effects, regardless of the treatment arm effect, are depicted in Table S3. For depression, being female compared to male was associated with a significant reduction in depression scores (*ß* = −.161; 95% CI = −0.23 to −0.09, *p* < .001), while being able to write was also associated with a decrease in depression (*ß* = −.092; 95% CI = −0.18 to −0.01, *p* = .033). Regarding anxiety, being female compared to male was linked to a significant increase in anxiety (*ß* = .077; 95% CI = 0.00–0.15, *p* = .045). Additionally, living in Koinadugu compared to Kailahun was significantly associated with lower scores on all outcomes (meaning ‘better’ scores), namely emotion regulation (*ß* = −.276 = 95% CI = −0.38 to −0.18, *p* < .001), depression (*ß* = −.078; 95% CI = −0.16 to 0.00, *p* = .054), anxiety (*ß* = −.130; 95% CI = −0.22 to −0.04, *p* = .005), anxiety/depression (*ß* = −.188; 95% CI = −0.28 to −0.09, *p* < .001), and functional impairment (*ß* = −.144; 95% CI = −0.23 to 0.05, *p* = .002). Kono versus Kailahun was associated with lower scores on emotion regulation (*ß* = −.184; 95% CI = −0.29 to −0.08, *p* < .001) and with lower scores on anxiety/depression (*ß* = −.127; 95% CI = −0.23 to −0.03, *p* = .012), but not on other outcomes.

To explore the mediation effect of COVID‐19 burden (i.e., economic stressors and changed family relationships due to COVID‐19) on the relationship between study arm and mental health outcomes over time we focused exclusively on models where a relationship was observed between the combined effects of study arm and time with mental health outcomes (i.e., depression symptoms and combined depression and anxiety symptoms). The inclusion of COVID‐19 economic stressors in the model resulted in a minimal change in the coefficient of the study arm by time point interaction term, from *ß* = −.053 to *ß* = −.055, indicating that the effect of this interaction on depression symptoms remained relatively stable even after accounting for economic stressors related to the COVID‐19 pandemic. Results from the structural equation modeling (SEM) analysis revealed that COVID‐19 economic stressors mediated the relationship between the study arm by time point interaction and depression symptoms, with the interaction effect on COVID‐19 economic stressors being significant (*ß* = .009, *SE* = .004, *p* = .018). Additionally, COVID‐19 economic stressors had a direct positive effect on depression symptoms (*ß* = .015, *SE* = .004, *p* = .001), while the direct negative effect of the study arm by time point interaction on depression symptoms remained statistically significant (*ß* = −.036, *SE* = .002, *p* < .001). Conversely, the inclusion of changed relationships due to COVID‐19 did not result in any change in the coefficient, and was therefore not further investigated using SEM.

## Discussion

The present study demonstrates that the combined Youth Readiness Intervention (YRI) and entrepreneurship program led to prolonged positive effects on the mental health of Sierra Leonean youth receiving the intervention 4 years later. These results are in line with prior research on the YRI delivered within Sierra Leonean schools (Betancourt et al., 2014). Although long‐term effects were less pronounced, YRI + entrepreneurship program participants showed statistically significant reductions in depression and combined anxiety‐depression symptoms compared to controls at a longer follow‐up than previously reported. There were no differences evident in positive mental health effects for entrepreneurship program only participants (with no YRI) versus YRI + entrepreneurship participants, as found in a previous YRI effectiveness study (Freeman et al., 2024).

Our study highlights positive long‐term mental health effects of integration of the evidence‐based YRI into existing youth entrepreneurship programs in Sierra Leone, demonstrating their potential for sustained, longer‐term impact. Building on previous YRI work (Freeman et al., 2024) and current findings, it is likely that the return on investment of integrating the YRI for nonspecialist delivery as integrating the YRI for nonspecialists within entrepreneurship programs is even greater than reported previously, given outcomes observed at 4 years of follow‐up. Sustained improvements in mental health may reflect the high implementation fidelity and facilitator competence documented during the initial Youth FORWARD trial (Freeman et al., 2024). Implementation barriers identified during scale‐out were addressed through the Collaborative Team Approach (CTA) guided by the EPIS framework, as detailed in (Freeman et al., 2024). While the present analyses did not test mechanisms of barrier resolution, the high facilitator fidelity, competence, and iterative quality‐improvement processes documented during implementation likely supported the sustained effectiveness of the combined YRI + ENTR intervention—reflecting the intended integration of implementation and effectiveness within the Hybrid Type II design.

The sustained effects are especially noteworthy given Sierra Leone's challenging sociopolitical context—marked by a legacy of civil war, political instability, and recurrent crises such as Ebola, COVID‐19, and climate disasters. Youth face high unemployment, poverty, and limited access to education, while mental health services remain scarce and stigma persists (World Health Organization, 2018; Yu et al., 2023). Looking ahead, the EPIS framework offers guidance on how the YRI might be better sustained and scaled by working within alternate delivery platforms, including through digital platforms (e.g., WhatsApp‐based check‐ins), mobile‐based training for nonspecialists (e.g., video‐based applications), and integration into educational or vocational programming. These findings highlight the feasibility of embedding mental health interventions within existing government systems and underscore the need for continued collaboration among policymakers and implementers to sustain delivery and local adaptation.

The sustained effects observed in Sierra Leone are consistent with evidence from other countries where the YRI has been implemented or adapted to address the needs of conflict‐affected youth. In Colombia, the YRI was culturally adapted and piloted among youth affected by armed conflict, demonstrating significant improvements in emotion regulation and functioning (Pineros‐Leano et al., 2024). Similarly, in Rwanda, where elevated levels of psychological distress have been documented among postgenocide youth (Kayiteshonga, Sezibera, Mugabo, & Iyamuremye, 2022), the integration of scalable, evidence‐based mental health interventions within existing service platforms has been identified as a national priority. In this context, school‐based YRI delivery through existing educational structures represents a promising integration model. Together, these examples underscore the YRI's flexibility and potential for scale across diverse low‐resource and postconflict contexts, supporting the global relevance of the present findings.

We also observed significant district‐level differences in outcomes, with participants in Koinadugu and Kono reporting lower distress compared to those in Kailahun. These patterns likely reflect differences in postconflict recovery, infrastructure, service availability, and community stress (Horn, Arakelyan, Wurie, & Ager, 2021). Kailahun continues to face significant challenges, including limited services and high levels of distress, which may hinder youth engagement and their response to the combined YRI and entrepreneurship intervention (Hopwood et al., 2021). In contrast, Koinadugu and Kono may have offered relatively more stable or supportive environments, which could enhance coping or reduce the overall symptom burden. While these district effects were statistically adjusted for in the mixed‐effects models and therefore are unlikely to represent confounding in the estimation of the impact of the combined YRI and entrepreneurship intervention, they nonetheless highlight the importance of considering local context when interpreting findings and designing scalable interventions (Freeman et al., 2024). Future studies should further investigate how regional differences in exposure to adversity, service infrastructure, and cultural norms influence mental health and responsiveness to the combined intervention.

In line with previous YRI research (Betancourt et al., 2014; Freeman et al., 2024), we found no sustained improvements for daily functioning and emotional regulation for YRI plus entrepreneurship participants compared to those in the control arm. This could potentially be due to several external factors, such as economic hardship or COVID‐19‐related disruptions. In contexts where meeting basic needs is a challenge, improvements in psychological well‐being may not readily translate into improvements in functional or emotional regulation (Jailobaeva et al., 2022). That said, the combined YRI and entrepreneurship intervention still produced measurable improvements in mental health outcomes, particularly anxiety and depression. This suggests that these domains may be more immediately responsive to the program's focus on emotional regulation and trauma‐related psychoeducation. Further research is needed to explore how to better target youth’ daily functioning and emotional regulation, particularly in fragile and postconflict settings, such as Sierra Leone, where external stressors are significant.

One of these external stressors that were studied were COVID‐19‐related economic stressors. These stressors partially explained the relationship between study arm, time, and depression or combined anxiety‐depression symptoms. While effects were small, they highlight the pervasive impact of pandemic‐related stress on mental health. These stressors affected all participants similarly, potentially diluting intervention‐specific effects. Nonetheless, the combined YRI and entrepreneurship intervention's positive impact on depression remained robust, suggesting its effects were resilient to external economic stress. The lack of an observed effect of COVID‐19‐related relationship strain on mental health outcomes may be due to the possibility that deteriorated relationships are not a precursor to mental health problems, but rather a consequence of them (Pieh, O´ Rourke, Budimir, & Probst, 2020). Since COVID‐19 variables were assessed concurrent with the four‐year follow‐up and reported retrospectively, additional research is needed to assess the temporal order with mental health outcomes.

Study limitations must be noted. Findings may not be generalizable to youth in more urban areas of Sierra Leone as the sample was predominantly rural, older, and had a lower educational level. The age range (18–30 years) reflects the Government of Sierra Leone's definition of ‘youth,’ which differs from international standards such as those of the WHO and United Nations (15–24 years). However, this broader definition allowed us to capture a population segment that remains highly relevant to national youth programming and policy priorities. Additionally, while effect sizes of .1–.2 may seem clinically modest, given the small sample size and the long follow‐up period of four years, these effect sizes are noteworthy, and if extrapolated to a larger population, these effects could prove to be substantial, cf. (Attanasio, Meghir, & Santiago, 2012). Comparable small but meaningful effects were observed in the same population at immediate postintervention follow‐up (Freeman et al., 2024), where reductions in depression (effect size = .15) and anxiety (effect size = .08) were detected for the combined YRI and entrepreneurship group relative to controls. Earlier YRI trials in different youth populations and using different measures similarly reported small‐to‐moderate short‐term effects (effect sizes ranging from −.32 to .31) on emotion regulation, prosocial behavior, and functional impairment (Betancourt et al., 2014). When viewed alongside other mental health interventions in low‐ and middle‐income countries—which typically yield small average effects (.20–.30) across psychological outcomes sustained beyond 1 year (Mendelson & Eaton, 2018; Patel et al., 2018; Singla et al., 2017)—the effects observed here are consistent with the broader evidence. These findings indicate that small effect sizes are not unexpected in such contexts and that the sustained impacts observed here align with other LMIC interventions. The ripple effects of improved mental health at the community level—on youth families, their peer groups, and also on the broader Sierra Leonean society—could further amplify these benefits. Also, an important aspect of the original Hybrid Type 2 Youth FORWARD study design was a strategy aimed at enhancing the delivery of evidence‐based interventions by nonspecialists (CTA) in a country where specialized mental health services are scarce. This enabled the scaling of mental health interventions to broader populations despite limited resources. Thus, while the treatment effect sizes in this study may be small, the success of the CTA when the intervention was delivered demonstrated that even modest improvements can have a meaningful impact on the well‐being of youth 4 years later.

Future research is needed to investigate whether the short‐term benefits related to labor market outcomes, such as increased income among YRI + entrepreneurship program participants, persist 4 years later. This would examine how, if present, these sustained benefits are related to participants' mental well‐being. Given the absence of significant differences between entrepreneurship‐only and YRI + entrepreneurship program participants, qualitative exploration of participants' experiences with the YRI + ENTR could provide insights into distinct contributions and outcomes of the YRI and ENTR components. Future studies should also examine whether this combination produces synergistic or additive effects beyond either component alone. Additionally, future YRI studies will explore diffusion and spillover effects, examining whether YRI benefits extend to others in participants' social networks and caregivers. Recent analyses have quantified the economic impact of integrating the YRI with entrepreneurship programming. (Bowser et al., 2025) reported positive returns on investment, indicating that the combined YRI + ENTR model can generate meaningful economic and health‐related benefits. Finally, given the new challenges posed by the ‘Kush’ epidemic in Sierra Leone—a rapidly spreading synthetic drug associated with cognitive, behavioral, and physical health deterioration among youth—future research should also explore its interplay with mental health outcomes (Eze et al., 2024; Lahai, Vandy, Turay, Kolipha‐Kamara, & Conteh, 2025). This calls for an adaptation of existing evidence‐based programs like the YRI to integrate substance use prevention components that address emerging risks of substance abuse but also strengthen youths' knowledge, attitudes, self‐efficacy, and behavioral skills related to substance use and mental health.

## Conclusion

In low‐income countries like Sierra Leone, where formal mental health services are limited, sustainable community‐based interventions that integrate mental health support with entrepreneurship programming offer a vital approach to addressing psychological distress. The sustained benefits suggest that participants have developed internal resources and coping strategies that help them navigate ongoing environmental challenges, including major disruptions like the COVID‐19 pandemic. These lasting gains may also contribute to broader community resilience, creating ripple effects that strengthen both individual well‐being and collective capacity to adapt to future adversity. Beyond its local relevance, this study contributes to the field of child psychology and psychiatry by extending evidence on the long‐term effectiveness of a transdiagnostic, group‐based intervention for youth in a low‐resource, postconflict context, underscoring the importance of culturally grounded and sustainable approaches to promoting youth mental health globally.

## Ethical considerations

All participants provided oral informed consent prior to eligibility screening and enrollment. This study was conducted in accordance with the Declaration of Helsinki. Ethical approval was obtained from the Boston College Institutional Review Board (Protocol #203.099.01; approved April 22, 2022) and the Sierra Leone Ethics and Scientific Review Committee (approval granted May 18, 2022; no protocol number issued).

**Key points**

**What's known?**

- In Sierra Leone, where formal mental health services are limited, the combined Youth Readiness Intervention (YRI) and entrepreneurship program has shown promise in improving youth mental health through community‐based delivery.

**What's new?**

- This study provides evidence that the combined YRI and entrepreneurship program is associated with sustained mental health benefits, even in the face of ongoing adversity such as the COVID‐19 pandemic, although no significant improvements were observed for daily functioning or emotion regulation outcomes.

**What's relevant?**

- Findings underscore the value of scalable, community‐delivered mental health programs in building individual and community resilience where formal mental health systems are limited.

## Supporting information


**Table S1.** Means, standard errors, 95% intervals, and group size for clinical effectiveness outcomes (from imputed data).
**Table S2**. Youth mental health four‐year follow‐up intervention effectiveness estimates from the mixed‐effects models.
**Table S3**. Results from the other fixed effects.

## Data Availability

The datasets used and/or analyzed during the current study are available from the corresponding author upon reasonable request.
