# Supplementary material for: Sustained mental health outcomes from the youth readiness intervention: A four‐year effectiveness follow‐up of a hybrid type II trial in Sierra Leone
Source: J Child Psychol Psychiatry. 2026 Apr 27;67(9):1537–46. doi: 10.1111/jcpp.70158 (PMC13450158; doi:10.1111/jcpp.70158)
Supplement: Supplementary file 1 [file JCPP-67-1537-s001.docx]

**Sustained Mental Health Outcomes from the Youth Readiness Intervention: A Four-Year Effectiveness Follow-Up of a Hybrid Type II Trial in Sierra Leone**

**Supporting Information**

**Table S1.** Means, Standard Errors, 95% Intervals, and Group Size for clinical effectiveness outcomes (from imputed data)

| Outcomes | **Baseline** | | | | |  | **Four years follow up** | | | | |
| --- | --- | --- | --- | --- | --- | --- | --- | --- | --- | --- | --- |
|  | Mean | Std. Error | 95% C.I. | | Group Size |  | Mean | Std. Error | 95% C.I. | | Group Size |
|  |  |  | Lower Bound | Upper Bound |  |  |  |  | Lower Bound | Upper Bound |  |
| **Difficulties in Emotion Regulation (DERS)** | |  |  |  |  |  |  |  |  |  |  |
| Control | 1.726 | 0.029 | 1.669 | 1.784 | 387 |  | 1.608 | 0.034 | 1.542 | 1.674 | 189 |
| ENTR Only | 1.740 | 0.028 | 1.684 | 1.795 | 380 |  | 1.559 | 0.031 | 1.497 | 1.620 | 201 |
| YRI + ENTR | 1.718 | 0.028 | 1.664 | 1.773 | 384 |  | 1.571 | 0.031 | 1.509 | 1.632 | 194 |
| **Depression (HSCL)** |  |  |  |  |  |  |  |  |  |  |  |
| Control | 1.741 | 0.028 | 1.686 | 1.796 | 387 |  | 1.591 | 0.031 | 1.529 | 1.652 | 189 |
| ENTR Only | 1.739 | 0.025 | 1.689 | 1.788 | 380 |  | 1.519 | 0.027 | 1.467 | 1.573 | 201 |
| YRI + ENTR | 1.829 | 0.029 | 1.772 | 1.887 | 384 |  | 1.525 | 0.027 | 1.472 | 1.577 | 194 |
| **Anxiety (HSCL)** |  |  |  |  |  |  |  |  |  |  |  |
| Control | 1.689 | 0.030 | 1.631 | 1.747 | 387 |  | 1.510 | 0.029 | 1.453 | 1.569 | 189 |
| ENTR Only | 1.667 | 0.064 | 1.615 | 1.719 | 380 |  | 1.423 | 0.026 | 1.371 | 1.719 | 201 |
| YRI + ENTR | 1.755 | 0.028 | 1.689 | 1.801 | 384 |  | 1.489 | 0.027 | 1.435 | 1.801 | 194 |
| **Anxiety and Depression Total Score (HSCL)** | | |  |  |  |  |  |  |  |  |  |
| Control | 1.714 | 0.027 | 1.661 | 1.767 | 387 |  | 1.561 | 0.028 | 1.505 | 1.616 | 189 |
| ENTR Only | 1.709 | 0.024 | 1.662 | 1.755 | 380 |  | 1.478 | 0.024 | 1.430 | 1.524 | 201 |
| YRI + ENTR | 1.788 | 0.028 | 1.734 | 1.842 | 384 |  | 1.511 | 0.025 | 1.462 | 1.556 | 194 |
| **Functional Impairment (WHODAS)** |  |  |  |  |  |  |  |  |  |  |  |
| Control | 0.279 | 0.024 | 0.233 | 0.327 | 387 |  | 0.252 | 0.024 | 0.203 | 0.300 | 189 |
| ENTR Only | 0.261 | 0.020 | 0.221 | 0.299 | 380 |  | 0.192 | 0.023 | 0.147 | 0.238 | 201 |
| YRI + ENTR | 0.299 | 0.026 | 0.245 | 0.350 | 384 |  | 0.228 | 0.022 | 0.183 | 0.272 | 194 |

Note. 95% C.I. = 95% Confidence Interval; DERS = Difficulties in Emotion Regulation Scale; HSCL=Hopkins Symptom Checklist; WHODAS = World Health Organization Disability Adjustment Scale.

**Table S2.** Youth mental health four-year follow-up intervention effectiveness estimates from the mixed-effects models

| **Freeman et al*:** baseline – 2 months follow up | | | | |  | **Current study:** baseline – 4 years follow up | | | |
| --- | --- | --- | --- | --- | --- | --- | --- | --- | --- |
|  | *β* | 95%CI | *d* | *p* |  | *β* | 95%CI | *d* | *p* |
| **Difficulties in Emotion Regulation (DERS)** | | |  |  |  |  |  |  |  |
| ENTR vs Control | -0.041 | -0.093 – 0.009 | -0.073 | 0.112 |  | -0.019 | -0.06 - 0.02 | -0.036 | 0.332 |
| ENTR+YRI vs Control | -0.069 | -0.078 – 0.024 | -0.048 | 0.299 |  | -0.016 | -0.06 - 0.02 | -0.031 | 0.419 |
| ENTR+YRI vs ENTR | -0.015 | -0.036 – 0.066 | -0.025 | 0.577 |  | 0.003 | -0.04 - 0.04 | 0.006 | 0.872 |
| **Depression (HSCL)** |  |  |  |  |  |  |  |  |  |
| ENTR vs Control | -0.042 | -0.085 – 0.001 | -0.080 | 0.057 |  | -0.025 | -0.06 - 0.01 | -0.049 | 0.177 |
| ENTR+YRI vs Control | -0.081 | -0.124 – -0.038 | -0.154 | <0.001 |  | -0.057 | -0.09 - -0.02 | -0.111 | 0.002 |
| ENTR+YRI vs ENTR | -0.039 | -0.083 – 0.004 | -0.074 | 0.077 |  | -0.032 | -0.07 - 0.00 | -0.063 | 0.082 |
| **Anxiety (HSCL)** |  |  |  |  |  |  |  |  |  |
| ENTR vs Control | -0.028 | -0.070 – 0.013 | -0.054 | 0.188 |  | -0.020 | -0.06 - 0.02 | -0.039 | 0.293 |
| ENTR+YRI vs Control | -0.043 | -0.091 – 0.005 | -0.082 | 0.045 |  | -0.033 | -0.07 - 0.00 | -0.062 | 0.088 |
| ENTR+YRI vs ENTR | -0.015 | -0.057 - 0.027 | -0.028 | 0.497 |  | -0.013 | -0.05 - 0.02 | -0.025 | 0.505 |
| **Anxiety and Depression Total Score (HSCL)** | | |  |  |  |  |  |  |  |
| ENTR vs Control | -0.037 | -0.076 – 0.003 | -0.073 | 0.110 |  | -0.026 | -0.06 - 0.01 | -0.055 | 0.128 |
| ENTR+YRI vs Control | -0.065 | -0.105 – -0.026 | -0.130 | 0.001 |  | -0.047 | -0.08 - -0.01 | -0.096 | 0.007 |
| ENTR+YRI vs ENTR | -0.029 | -0.069 – 0.011 | -0.058 | 0.156 |  | -0.021 | -0.05 - 0.01 | -0.044 | 0.225 |
| **Functional Impairment (WHODAS)** | | |  |  |  |  |  |  |  |
| ENTR vs Control | -0.041 | -0.087 – 0.005 | -0.084 | 0.078 |  | -0.013 | -0.04 - 0.02 | -0.031 | 0.421 |
| ENTR+YRI vs Control | -0.036 | -0.081 – 0.009 | -0.075 | 0.118 |  | -0.016 | -0.05 - 0.01 | -0.038 | 0.299 |
| ENTR+YRI vs ENTR | -0.005 | -0.041 – 0.050 | 0.009 | 0.838 |  | -0.004 | -0.03 - 0.03 | -0.009 | 0.810 |

Note. DERS = Difficulties in Emotion Regulation Scale; HSCL=Hopkins Symptom Checklist; WHODAS = World Health Organization Disability Adjustment Scale. *Source: Freeman et al (2024). Integrating youth readiness intervention and entrepreneurship in Sierra Leone: A hybrid type II cluster randomized trial. Journal of the American Academy of Child & Adolescent Psychiatry, 63(7), 708-719.

**Table S3.** Results from the other fixed effects

|  | **DERS** | | |  | | **Depression** | | |  | | **Anxiety** | |  |
| --- | --- | --- | --- | --- | --- | --- | --- | --- | --- | --- | --- | --- | --- |
|  | *β* | ci95 | *p* | | *β* | | ci95 | *p* | | *β* | | ci95 | *p* |
| Age | -0.002 | -0.01 - 0.00 | 0.507 | | 0.000 | | -0.01 - 0.01 | 0.903 | | 0.006 | | -0.00 - 0.01 | 0.118 |
| Female vs male | -0.023 | -0.11 - 0.06 | 0.576 | | -0.161 | | -0.23 - -0.09 | 0.000 | | 0.077 | | 0.00 - 0.15 | 0.045 |
| Primary/beyond vs None/Some Primary* | 0.018 | -0.05 - 0.09 | 0.610 | | 0.015 | | -0.04 - 0.07 | 0.617 | | -0.018 | | -0.09 - 0.05 | 0.614 |
| Can read vs cannot read | 0.006 | -0.10 - 0.11 | 0.910 | | 0.044 | | -0.04 - 0.13 | 0.325 | | 0.065 | | -0.04 - 0.17 | 0.220 |
| Can write vs cannot write | -0.041 | -0.14 - 0.06 | 0.418 | | -0.092 | | -0.18 - -0.01 | 0.033 | | -0.030 | | -0.13 - 0.07 | 0.567 |
| Koinadugu vs Kailahun | -0.276 | -0.38 - -0.18 | 0.000 | | -0.078 | | -0.16 - 0.00 | 0.054 | | -0.130 | | -0.22 - -0.04 | 0.005 |
| Kono vs Kailahun | -0.184 | -0.29 - -0.08 | 0.000 | | -0.006 | | -0.09 - 0.08 | 0.883 | | -0.049 | | -0.14 - 0.04 | 0.304 |

|  | Anxiety/Depression | |  | WHODAS | |  |
| --- | --- | --- | --- | --- | --- | --- |
|  | *β* | ci95 | *p* | *β* | ci95 | *p* |
| Age | -0.003 | -0.01 - 0.00 | 0.396 | 0.002 | -0.00 - 0.01 | 0.567 |
| Female vs male | 0.050 | -0.03 - 0.13 | 0.215 | 0.065 | -0.01 - 0.14 | 0.085 |
| Primary/beyond vs None/Some Primary* | -0.000 | -0.07 - 0.07 | 0.990 | -0.017 | -0.08 - 0.05 | 0.609 |
| Can read vs cannot read | 0.054 | -0.05 - 0.16 | 0.309 | 0.063 | -0.03 - 0.16 | 0.202 |
| Can write vs cannot write | -0.029 | -0.13 - 0.07 | 0.574 | -0.034 | -0.13 - 0.06 | 0.480 |
| Koinadugu vs Kailahun | -0.188 | -0.28 - -0.09 | 0.000 | -0.144 | -0.23 - -0.05 | 0.002 |
| Kono vs Kailahun | -0.127 | -0.23 - -0.03 | 0.012 | -0.073 | -0.17 - 0.02 | 0.120 |

Note. * Completed educational level. DERS = Difficulties in Emotion Regulation Scale; WHODAS = World Health Organization Disability Adjustment Scale. Depression and anxiety were measured with the Hopkins Symptom Checklist.
